# Supplementary material for: Identification of photoperiod-induced specific miRNAs in the adrenal glands of Sunite sheep (Ovis aries)
Source: Front Vet Sci. 2022 Jul 22;9:888207. doi: 10.3389/fvets.2022.888207 (PMC9354845; doi:10.3389/fvets.2022.888207)
Supplement: Supplementary file 1 [file Data_Sheet_1.ZIP › Supplementary file/miRDeep2 file.zip!/miRDeep2 file/Know_Structure/LP42A3/oar-mir-299.pdf]

miRBase precursor : oar-mir-299
Total read count : 343
oar-miR-299-5p read count : 335
oar-miR-299-3p read count : 7
remaining reads : 1

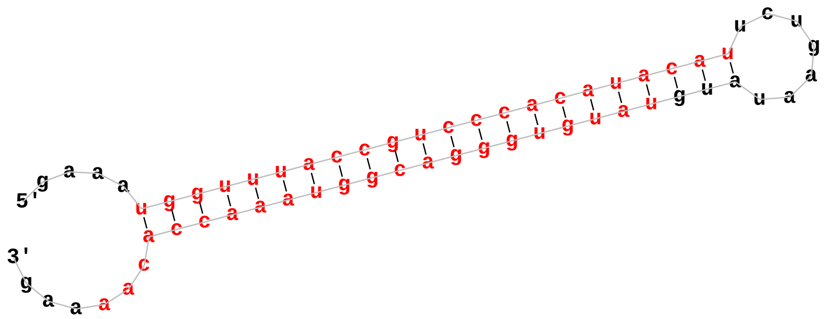

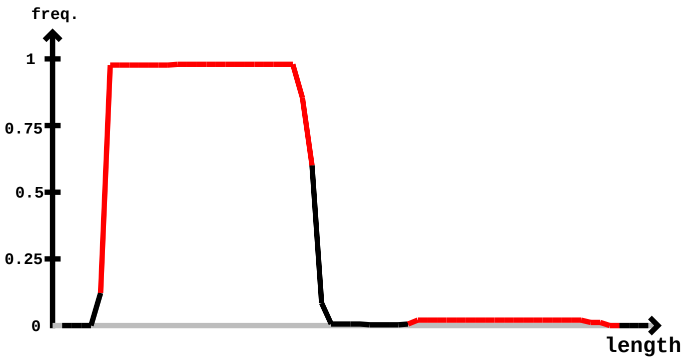


```
       oar-miR-299-5p
                                          oar-miR-299-3p
5'- gaaaugguuuaccgucccacauacauucugaauauguaugugggacgguaaaccacaaaag   -3'    exp
    ....((((((((((((((((((((((.......))))))))))))))))))))))......    reads      mm         sample
    ...augguuuaccgucccacauac.....................................    8          0          seq
    ...augguuuaccgucccacauaca....................................    33         0          seq
    ...augguuuaccgucccacauacau...................................    1          0          seq
    ....ugguuuaccgucccacauac.....................................    35         0          seq
    ....ugguuuaccgucccacauaca....................................    54         0          seq
    ....ugguuuaccgucccacauacau...................................    176        0          seq
    ....ugguuuaccgucccacauacauA..................................    6          1          seq
    ....ugguuuaccgucccacauacauu..................................    21         0          seq
    ....ugguuuaccgucccacauacauucugaaua...........................    1          0          seq
    ...........ccgucccacauacauucuga..............................    1          0          seq
    ..................................uguaugugggacgguaaacc.......    1          0          seq
    ...................................guaugugggacgguaaaccac.....    1          0          seq
    ....................................uaugugggacgguaaacc.......    2          0          seq
    ....................................uaugugggacgguaaaccac.....    3          0          seq
```
